# The No-Destination Ship of Priority-Setting in Healthcare: A Call for More Democracy

**DOI:** 10.15171/ijhpm.2017.119

**Published:** 2017-10-11

**Authors:** Brayan V. Seixas

**Affiliations:** School of Population and Public Health, University of British Columbia, Vancouver, BC, Canada.

**Keywords:** Healthcare Rationing, Public Participation, Democracy, Organizational Decision-Making

## Abstract

In dealing with scarcity of resources within healthcare systems, decision-makers inevitably have to make choices about which services to fund. Setting priorities represents a challenging task that requires systematic, explicit and transparent methodologies with focus on economic efficiency. In addition, the engagement of the general public in the process of decision-making has been regarded as one of the most important aspects of the management of publicly-funded health systems in liberal democracies. In the current essay, we aim to discuss the problematics of public engagement in the process of resource allocation and priority-setting within the context of publiclyfunded health systems. Our central argument is that although there may be a conflict between democratic mechanisms of citizen participation and economic efficiency, in the extra-welfarist sense, expected for/from the system, the solution for this tension does not seem to rely on more or novel authoritative technocratic approaches, but rather on the deepening and betterment of democratic participation.

“Utopia is on the horizon. I move two steps closer; it moves two steps further away. I walk another ten steps and the horizon runs ten steps further away. As much as I may walk, I’ll never reach it. So what’s the point of utopia? The point is this: to keep walking.”^1^ *Eduardo Galeano*

## Introduction

In dealing with scarcity of resources in the healthcare system, decision-makers inevitably have to make choices about which services to fund (and, of course, which services not to fund). Determining priorities represents a challenging task that arguably requires systematic, explicit and transparent methodologies with focus on efficiency. Moreover, publicly-accountable decisions and mechanisms of democratic participation in the process of decision-making have also been defended as fundamental parts of fair and high-performance management of public healthcare systems in liberal democracies.^2^

In the current essay, we aim to discuss the problematics of engaging the public in the process of resource allocation and priority-setting within the context of publicly-funded healthcare systems, with a particular focus on the widest levels of governance. Our central argument is that although there may be a conflict between democratic mechanisms of citizen participation and economic efficiency expected for/from the system, the solution for this tension does not seem to rely on more or novel authoritative technocratic approaches, but rather on the deepening and betterment of democratic participation.

## 2. Contextualization

Every health system in the world, in high-income countries or not, has to deal with the problem of scarcity. As Mitton and Donaldson point out,^2^ “scarcity is here to stay,” for the claims on healthcare procedures will always be greater than the actual resources at hand. Aging populations and the ever-increasing technological innovations in healthcare are commonly referred to as the main reasons for the unsustainable force acting on healthcare systems worldwide.

In the province of British Columbia (Canada), for instance, expenditures on healthcare have risen at an astonishingly unsustainable rate. In the late 1990s, roughly 33% of the provincial budget was used with healthcare. This figure is currently around 43% and estimates indicate that it will reach 52% by 2030.^3^ Despite this trend, there is still a continuous perception that many healthcare demands are unmet and, consequently, more resources would be necessary.

Within this scenario, there is no easy solution. Policy-makers, care-providers and citizens have to understand that scarcity is here to stay and that we, as society, need to develop means of tackling this paramount issue. Thus, setting priorities for investment becomes a central concern of health organizations in general.

Multiple strategies have been used and suggested to determine investment priorities in a systematic and explicit way,^2^ from non-economic ones (such as needs assessment and core services definition) to economic methods, including different types of economic evaluation, quality-adjusted life year (QALY) league tables, program budgeting and marginal analysis (PBMA) and multi-criteria decision analysis (MCDA). Each of these methods relies on different ethical, economic and managerial principles to deal with the problem of decision-making in priority-setting and they contemplate the issue of public engagement in different manners.

## 3. The Notion of Economic Efficiency in the Context of Health Systems

In the field of neoclassical economics, the term efficiency commonly encompasses three major dimensions. Technical efficiency approaches the issue of efficiency in terms of the production of goods (productivity) and entails searching for all the possible means to achieving the highest possible outputs for a given amount of inputs. Cost-effectiveness efficiency basically seeks to determine the least costly method of production among the technically efficient ones. The final dimension—allocative efficiency—deals with the distribution of goods in relation to the way individuals value and judge these goods. This broader and more complex notion of efficiency takes into account individual preferences, ie, the utility derived from goods or services.

Within the realm of health economics, there are two major paradigms dealing with the notion of allocative efficiency: welfarism and extra-welfarism.^4^ For the former, the allocation of resources should be carried out in a fashion that maximizes the overall sum of individual utilities as long as the gains of the winners are sufficiently large to compensate the losers for their losses and be still better off (the Potential Pareto Improvement principle). In the welfarist universe, health services are understood in the same way as any other goods and therefore their utility is derived from their consumption. The corollary, thus, is that the utility of health services is not health itself (or more health), but the values that consumers attribute to health services. Within this context, the maximization of individual utilities maximizes overall efficiency.

The more prevailing paradigm, though, seems to be the extra-welfarist approach, which advocates that the ultimate role of health systems is to increase the overall health of the society and that this principle, applied through cost-effectiveness analysis, should guide decision-making about which investments shall be made. Several indicators of health have been developed to numerically translate this concept, and provide practical ways of analysing which practices and technologies lead to the maximization of overall health. In this regard, the most widely used indicator is QALYs, a combination of years and quality of life gained through an intervention.

In the next section, we will discuss how the pursuit for economic efficiency may conflict with the idea of democratic participation in the processes of decision-making in resource allocation and priority-setting. Any suggestion that economic efficiency and public engagement are inevitably in conflict is far too simple; we aim instead to shed light onto subtle but important nuances around this topic.

## 4. The Challenging Endeavor of Engaging the Public in Resource Allocation Processes

Democracy constitutes a fundamental value for the modern western world. The extent of its discursive power is so vast that very often it seems to represent a sort of holy grail for modern industrialized societies. However, a variety of criticisms against democracy have been put forward for centuries, surely at least since Plato. For him, democracy as a system of governance is necessarily inefficient, because society is ruled by non-experts.^5^ In his view, every decision concerning the “*res publica*” (literally the ‘public things’) should be determined by the individuals with wisdom and specialized knowledge on the given subject.

Going back to modern healthcare systems, this tension between experts and non-experts recurs as a common dilemma. Engaging the public is repeatedly argued as an essential means of carrying out an accountable process of decision-making, especially when difficult choices have to be made. On the other hand, though, bringing non-specialized voices to the process may indeed imply a loss of efficiency.

Although there is no clear and consensual definition of democracy among political theorists, for the purpose of situating our discussion, we work with the minimalistic definition provided by Noberto Bobbio: “a ‘democratic regime’ is taken to mean first and foremost a set of procedural rules for arriving at collective decisions in a way that accommodates and facilitates the fullest possible participation of interested parties.”^6^

When it comes to priority-setting in the healthcare system,^7^ lack of technical expertise in topics such as health economics concepts, population health perspectives and elements of biomedical sciences, is only one of many problems. Rice^8^ points out that “individuals need to be protected from their own foolishness.” Although this may sound an arrogant stance of an individual who seemingly knows what is best for others, this paternalistic position may be really required if the society truly cares more about its overall health than the perceived interests of its citizens. The anti-vaccination movement is a clear example of this potential problem. Its defenders prefer not to vaccine themselves or their children, although that attitude puts the whole population at risk. Another illustration is the exercise of quarantine during outbreaks of some infectious disease. Individuals might actually not value this practice and; yet the non-isolation of infected persons may lead to huge negative impacts for all. Consequently, we can conclude that the maximization of welfare in the health sector is not necessarily achievable through the consideration of individuals’ values or interests.

Even when individuals do not act “foolishly” or against their own health status, they might possess an “inability to desire,” as pointed out by Amartya Sen.^9^ His argument is that the preferences and interests people carry with them are not intrinsic expressions of individual selves. Our desires are shaped by the experiences and opportunities of the social environments in which we have been raised and live. People within the lower social ranks have truncated expectations, conditioned by a lifetime (or even generations) of restricted possibilities. “Their horizon of well-being is likely narrowed by the embodiment of the disadvantaged social position.”^4^

Another barrier to meaningful public engagement in healthcare resource allocation is that the public usually receives a skewed set of information about priorities in healthcare. Mass media vehicles tend to focus on cutting-edge health technologies and emotionally moving subjects, with little coverage of the benefits prevention, health promotion and management of the social determinants of health. Furthermore, it is pertinent to remember the long list of cognitive biases that lead us to “misinterpret” information and take “irrational” choices, or put in a different way, all the common psychological devices that flaw our desired/expected rationality as decision-makers.^10^

While many economists genuinely believe that the overall efficiency of allocation can be achieved through the overall sum of individual values (or to lapse into technical jargon, utilities), the arguments elaborated here demonstrate that this rationale does not hold true in the context of health systems. This raises the question: should we abandon this obsession for democracy as a core principle in favour of efficiency as the most important criterion for healthcare resource allocation decisions?

In order to answer this inquiry, let us start by quoting Churchill “*No one pretends that democracy is perfect or all-wise. Indeed, it has been said that democracy is the worst form of Government except for all those other forms that have been tried from time to time.*”^11^ Above all, it is fundamentally important that we highlight that democracy has a value in itself. We, the so-called moderns, do not esteem democracy because of any potential benefit derived from it. Its value relies basically on the fact that it consubstantiates our desire for formal equality, dignity, freedom and autonomy.

Thus, our duty is to figure out ways of combining the pursuit of efficiency in resource allocation with the underlying democratic principle. This hitherto unresolved tension has attracted great thinkers for centuries and we have no naïve presumption of ending the debate, but offer insights to encourage active strategies of public involvement and to further the discussion of methodological possibilities.

## 5. More Democracy, not Less

Democracy is an underlying principle manifest in the form of a process, not a fully finished outcome itself. In this sense, decision-makers in the healthcare system must be cognizant of the challenges posed by public involvement in priority-setting and resource allocation in order to advance the process.

A variety of methodologies for public involvement in the process of priority-setting and resource allocation have been proposed,^12^ such as the direct participation of representatives in advisory/deliberative committees, referendums, opinion polls, citizen juries, focus groups, etc. Yet, a pan-Canadian survey^13^ conducted with decision-makers involved with the process of setting priorities in the realm of cancer control has found that only around half of them reported using strategies of public involvement “often” or “always.” Moreover, decision-makers reported that, among other barriers to public involvement, the most limiting one is that scientific evidence is valued over the evidence supplied by the public.

It is an astonishing revelation that decision-makers tend to believe that science has a role more important in the process of setting priorities than public preferences. At the end of the day, it is imperative to recall that science is not a neutral or value-free epistemology.^14^ No matter how consistent and supposedly objective is a scientific endeavour, science is always a social construct and, as such, necessarily embodies the values and limitations of its context of development. And even if we simply ignored this whole critique, we would still have to deal with the extreme levels of uncertainty in the medical field.

In addition, it is absolutely crucial to emphasize that there is no right way of allocating resources that, in principle, belong to everyone (in the case of publicly-funded systems); consequently, the process of decision-making is ultimately political, even though we may use ‘rationalistic’ methodologies to support it. Hence, here it is not possible to replace politics with science, and our duty as policy-makers and researchers is to pursue strategies to combine societal values, experts’ opinions and “hard” evidence in a methodology that leads us towards explicit, consistent, fair and accountable decisions.

Being by definition a process, the democratic endeavour of decision-making in the healthcare system needs constant evaluation. Once it is acknowledged that there are no consensual and immutable goals in the process of resource allocation, it is vital that assessment practices take place regularly. Engaging the public is a challenging task that requires continuous learning. The enormous barriers inherent to this process should by no means serve as an excuse for lack of action and for adhering to a technocratic discourse.

Coming back to the ancient roots of this philosophical debate, Plato utilized a metaphor that has retained its currency in political philosophy: the ship of state. Generally speaking, Plato said that democracy is a terrible system because if we represented the state by a ship, it would mean that, instead of leaving the ship in control of a knowledgeable and experienced sailor, we would be guided by non-experts fighting for power and, therefore, we would navigate aimlessly. However, for us moderns, democracy is not about getting to a certain place, but rather learning the process of being together in the ship, developing respect for the others and care for mutually important things (*res publica*).^15^ In the process of priority-setting and resource allocation in healthcare systems, our target must be the control of the ship in accordance with both scientific knowledge and societal values. As we progress in this quest for more democracy, the public will be more engaged and more informed. Thus, we will hopefully advance towards overall economic efficiency through public involvement, not despite it.

## Ethical issues

Not applicable.

## Competing interests

Author declares that he has no competing interests.

## Author’s contribution

BVS is the single author of the paper.
